# Bioinspired Silk Fibroin-Based Composite Grafts as Bone Tunnel Fillers for Anterior Cruciate Ligament Reconstruction

**DOI:** 10.3390/pharmaceutics14040697

**Published:** 2022-03-24

**Authors:** Viviana P. Ribeiro, João B. Costa, Sofia M. Carneiro, Sandra Pina, Ana C. A. Veloso, Rui L. Reis, Joaquim M. Oliveira

**Affiliations:** 1 3B’s Research Group, I3Bs—Research Institute on Biomaterials, Biodegradables and Biomimetics of University of Minho, Headquarters of the European Institute of Excellence on Tissue Engineering and Regenerative Medicine, AvePark, Parque de Ciência e Tecnologia, Zona Industrial da Gandra, Barco, 4805-017 Guimarães, Portugal; sandra.pina@i3bs.uminho.pt (S.P.); rgreis@i3bs.uminho.pt (R.L.R.); miguel.oliveira@i3bs.uminho.pt (J.M.O.); 2 ICVS/3B’s—PT Government Associate Laboratory, 4710-057 Braga, Portugal; 3 Instituto Politécnico de Coimbra (ISEC), Departamento de Engenharia Química e Biológica (DEQB), Rua Pedro Nunes, Quinta da Nora, 3030-199 Coimbra, Portugal; sofiacarneiro1997@gmail.com (S.M.C.); anaveloso@isec.pt (A.C.A.V.); 4 CEB—Centre of Biological Engineering, University of Minho, Campus de Gualtar, 4710-057 Braga, Portugal

**Keywords:** anterior cruciate ligament, tissue engineering, silk fibroin, osteointegration

## Abstract

Anterior cruciate ligament (ACL) replacement is still a big challenge in orthopedics due to the need to develop bioinspired implants that can mimic the complexity of bone-ligament interface. In this study, we propose biomimetic composite tubular grafts (CTGs) made of horseradish peroxidase (HRP)-cross-linked silk fibroin (SF) hydrogels containing ZnSr-doped β-tricalcium phosphate (ZnSr-β-TCP) particles, as promising bone tunnel fillers to be used in ACL grafts (ACLGs) implantation. For comparative purposes, plain HRP-cross-linked SF hydrogels (PTGs) were fabricated. Sonication and freeze-drying methodologies capable of inducing crystalline β-sheet conformation were carried out to produce both the CTGs and PTGs. A homogeneous microstructure was achieved from microporous to nanoporous scales. The mechanical properties were dependent on the inorganic powder’s incorporation, with a superior tensile modulus observed on the CTGs (12.05 ± 1.03 MPa) as compared to the PTGs (5.30 ± 0.93 MPa). The CTGs presented adequate swelling properties to fill the space in the bone structure after bone tunnel enlargement and provide a stable degradation profile under low concentration of protease XIV. The in vitro studies revealed that SaOs-2 cells adhered, proliferated and remained viable when cultured into the CTGs. In addition, the bioactive CTGs supported the osteogenic activity of cells in terms of alkaline phosphatase (ALP) production, activity, and relative gene expression of osteogenic-related markers. Therefore, this study is the first evidence that the developed CTGs hold adequate structural, chemical, and biological properties to be used as bone tunnel fillers capable of connecting to the ACL tissue while stimulating bone tissue regeneration for a faster osteointegration.

## 1. Introduction

Anterior cruciate ligament (ACL) is a dense connective tissue connecting the lateral femoral condyle and the tibial plateau required for the articular motion of the knee. It is mainly constituted of collagen type I, supporting the knee rotations without break. Indeed, the mechanical properties of ACL have an important influence in the performance of other tissues, including meniscus and osteochondral tissue, being affected by several external factors, such as activity level, age, and weight [1]. Orthopedic surgeons find in ACL repairing enormous challenges. The low healing properties, lack of vascularization and hierarchical structure of the collagen fibers are some of the factors that hinder effective and rapid ACL repair and reconstruction [2]. For this reason, autografts from patellar tendon and hamstring tendon have been the gold standard in ACL replacement. Advantages of these approaches include the vascularization, low immune response and tenocytes that are capable to differentiate in ligamentocytes [3]. Allografts can also be used as ACL substitutes, however, possible disease transmission and adverse immune responses are common problems associated with these implants [4]. All these factors have contributed to the search for improved tissue engineering (TE) approaches for ACL repair and reconstruction, represented by the 4 clinical trials currently registered on www.clinicaltrials.gov (Date of access: 14 February 2022; Filters: implant, grafts, anterior cruciate ligament) [5]. The use of synthetic materials has been quite explored with some market exploitation, e.g., Dacron^®^ (Kennesaw, GA, USA) and Gore-Tex^®^ (Newark, DE, USA) prostheses, or “Kennedy Ligament Augmentation Device^®^” (Kennedy LAD^®^, St. Paul, MN, USA) [6,7]. However, deformation and fracture limitations, low abrasion resistance, and lack of tissue regeneration are problems that strongly affect the use of such devices. On the other hand, natural polymers and proteins are less mechanically effective but more biocompatible, biodegradable and promotors of new tissue formation, which makes them possible substitutes for ACL replacement [5].

Regardless of the surgical approach, most implanted ACL grafts (ACLGs) become too large (>5 mm) and with low elastic recovery, which unable to obtain post-implantation grafts with similar properties to native ACL tissue [8]. Bone tunnel enlargement is also a recurrent phenomenon after grafts’ implantation affecting their long-term fixation [9]. Grafts’ micromotion within the tunnel, vibrations or defective fixation techniques are mechanically responsible for this occurrence [10]. The accumulation of intra-articular and synovial fluids in the space between the ACLGs and the bone wall may also cause local osteolysis and tunnel enlargement by the so-called “synovial bathing effect” [11]. In most scenarios, bone tunnel enlargement could be avoided by the inclusion of adequate fillers aligned with the implanted grafts, which would fulfill the tunnel space in the bone structure. This method would promote both stabilization and osteointegration of the grafts, accelerating bone tissue regeneration and soft tissue healing [2]. Previous studies proposed different calcium phosphate (CaP) cements, as structural bone tunnel fillers, to stabilize tendon grafts after implantation and to accelerate ACL reconstruction [12,13]. The results showed no significant improvements in comparison to the samples without bone fillers incorporation, suggesting that novel strategies should be considered to make the bone filling process more efficient. Recently, tissue engineers have been dedicated to the development of complex and hierarchical three-dimensional (3D) scaffolds for promoting tendon-to-bone healing [14]. For example, Park et al. [15] developed 3D bioprinted scaffold sleeves made of polycaprolactone (PCL), poly(lactic-co-glycolic acid) (PLGA) and β-tricalcium phosphate (β-TCP), and showed enhanced tendon-bone osteointegration in ACL reconstruction in a rabbit model. The biological healing was also promoted by the presence of mesenchymal stem cells (MSCs), which were induced in an early post-operative period, a secure biological healing between the two tissues. In a different approach, gelatin sponges embedded with platelet-rich plasma (PRP) were wrapped around the tendon graft and inserted into the bone tunnels of a rabbit ACL reconstruction model [15]. The success of this approach was demonstrated by the early healing process at the tendon-bone junctions, and by the prolonged bioactivity of PRP that stimulated MSCs osteoinduction. 

Silk fibroin (SF) is a particularly interesting biomaterial to be used in ACL tissue reconstruction, as it is the only protein that matches the required tensile properties of ACL, along with adequate biocompatibility and slow degradation for in vivo tissue in-growth and maturation [16]. In fact, the naturally derived SF is a source for scientific inspiration in several biomedical applications, presenting incredible biological mimicking features and healing capabilities [17]. The combination of SF with other polymers, such as PLGA, PCL or polyethylene terephthalate (PET) [18,19], in the form of fibrous/textile structures [20,21], has successfully been proposed for the fabrication of ACLGs. In fact, an ongoing clinical trial (NCT00490594) is validating the SeriACL^®^ device (AbbVie, Chicago, IL, USA) made of knitted silk to replace ACL and stabilize the knee joint after surgery (www.clinicaltrials.gov, date of access: 14 February 2022), with promising outcomes. An off-the-shelf approach was previously proposed by Li et al. [22] in which SF textile grafts were combined with a tricalcium phosphate (TCP)/polyether ether ketone (PEEK) anchor that served as supporting and osteoconductive material enhancing SF-based ACLG attachment to the bone tunnel space. Similarly, Shi et al. [23] developed SF meshes combined with low crystallinity hydroxyapatite (HAp) at both ends of the scaffolds promoting bone tunnel regeneration and osteoconductivity. In different studies, ACLGs made of knitted SF-collagen sponges were used as ACL substitutes for tissue reconstruction and specific ligament-bone healing [24,25]. Ligament-derived stem cells (LSPCs) were applied to the systems, wrapping the grafts as cell-sheets [24], or injected into the joint cavity of a rabbit model [25], providing abundant natural extracellular matrix (ECM) that facilitated ACL regeneration.

In the present study, new bone tunnel fillers were developed that are capable of improving ACLGs fixation and aiming to promote osteointegration while degrading. SF structures with tubular architecture were obtained from a horseradish peroxidase (HRP)-cross-linking method, which reacts with the tyrosine groups (≈5 mol%) on SF forming dityrosine cross-linking sites and covalently bound hydrogels (HRP-SF hydrogels). This in situ-cross-linkable system, i.e., at physiological conditions and with substrate-specific conjugation, has been deeply explored by our research group for applications that go from the development of engineering constructs for complex tissues regeneration, including cartilage [26], osteochondral [27,28,29], meniscus [30] and peripheral nerve [31], to 3D modeling of diseases [32]. By means of using the HRP-mediated cross-linking method the produced SF hydrogels are formed in a main random coil and transparent conformation, affecting the tyrosine groups on the amorphous and semi-crystalline regions of the SF protein [33]. These hydrogels are more biocompatible for cell encapsulation strategies and present suitable stability, which can undergo natural conformational transitions at macromolecular level over time, i.e., increase of β-sheet content, boosting their application for distinct TE strategies. In fact, the usually proposed SF hydrogels are formed through the transition of aqueous SF solutions from random coil to β-sheet under specific conditions, e.g., chemical and physical stimuli, such as ultrasonication, solvents adding, pH variations, and temperature/ion concentrations adjustments [34,35,36], which give the SF structures superior mechanical performance, stability and degradation properties [37,38,39]. Therefore, the potential of our work relies on the development of TGs formed by an HRP-mediated approach to cross-link SF combined with sonication and organic solvent system, towards producing robust and yet ductile TGs to make the bone filling process more efficient in ACLGs implantation. Moreover, the possibility of forming hybrid structures by the incorporation of β-TCP (ZnSr-β-TCP) doped with zinc (Zn) and strontium (Sr) into the SF hydrogel matrix represents the innovative character of the proposed composite TGs (CTGs), with enhanced mineralization properties and mechanical response for bone tunnel regeneration. From previous studies, it is well known that the presence of such ions into CaPs improves the bioactivity and influence the artificial ligaments osteointegration [27,40,41]. The physicochemical and mechanical characterization and suturability of the developed CTGs were assessed. The in vitro osteogenic behavior induced by this system was evaluated using human primary osteogenic sarcoma (SaOs-2 cells) cell line cultured up to 14 days. Plain TGs (PTGs) entirely composed of HRP-SF were also used as control. To the best of our knowledge, the combination of a processed HRP-SF hydrogel matrix and ion-doped β-TCP powders is herein proposed for the first time, aiming at faster bone regeneration in bone tunnel filling strategies and ACLGs osteointegration.

## 2. Materials and Methods

### 2.1. Materials and Reagents

The Portuguese Association of Parents and Friends of Mentally Disabled Citizens (APPACDM, Castelo Branco, Portugal) provided the *Bombyx mori* cocoons. All reagents were obtained from Sigma-Aldrich (St. Louis, MO, USA) unless otherwise described.

### 2.2. Preparation of the Tubular Grafts (TGs)

#### 2.2.1. Silk Fibroin (SF) Purification

Purified SF solution was obtained at high concentration (16 wt.%) from *Bombyx mori* cocoons, as previously described [26,27]. Briefly, silk cocoons were boiled in 0.02 M sodium carbonate solution for 1 h to remove the glue-like protein sericin and raised in abundant distilled water for the complete extraction of the degumming solution. The obtained SF was then dissolved in 9.3 M lithium bromide solution at 70 °C for 1 h, and dialyzed against distilled water for 48 h using benzoylated dialysis tubing (MWCO: 2 KDa). Next, the aqueous SF solution was concentrated in poly(ethylene glycol) for at least 6 h. The final concentration of SF as determined by the dry weight of a SF sample placed at 70 °C in the oven for at least overnight. In the meantime, the SF solution was stored at 4 °C until further use.

#### 2.2.2. Synthesis of ZnSr-Doped β-TCP Powders

Powders of β-TCP doped with 10 mol.% of Zn + Sr (ZnSr-β-TCP) were obtained by wet chemical precipitation, with a molar ratio of (Ca + Sr + Zn)/P = 1.48, as previously reported [42]. Briefly, calcium nitrate tetrahydrate [Ca(NO_3_).4H_2_O], diammonium hydrogen phosphate [(NH_4_)2HPO_4_], strontium nitrate [Sr(NO_3_)] and zinc nitrate [Zn(NO_3_)] were used as chemical precursors for Ca, P, Sr, and Zn, respectively. The precipitated suspension was maintained for 4 h under constant stirring and maturated in 8 M NH_4_OH solution for further 20 h in resting conditions at 50 °C and pH 7. The precipitate was vacuum filtered, dried at 100 °C, and heat treated for 2 h at 1100 °C. The obtained powders were milled and sieved in a mesh size of 36 μm, in order to obtain a controlled average particle size of 1–10 μm. 

#### 2.2.3. Preparation of HRP-SF/ZnSr-β-TCP TGs

**Figure 1:**
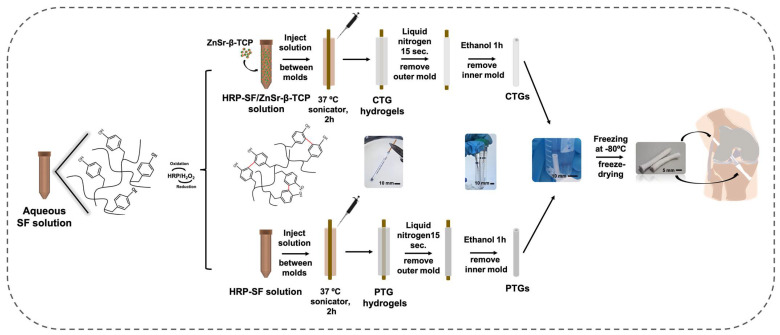
Schematic of the experimental setup used for preparing the CTGs, by processing the HRP-SF solution combined with the ZnSr-β-TCP powders in an 80/20 (*w*/*w*) ratio inside cylinder molds. HRP-SF solution processed alone was used to produce the PTGs used as control. Illustration shows the ultimate application of the CTGs as bone tunnel fillers in ACLGs implantation strategies.

An HRP-cross-linked SF solution (HRP-SF) was firstly prepared at 1/0.26‰/1.45‰ (SF/HRP/Hydrogen peroxide (H_2_O_2_)) [32], and then mixed with the ZnSr-β-TCP powders in a 80/20 (*w*/*w*) ratio (HRP-SF/ZnSr-β-TCP) by fast stirring for 2 min, followed by injection within the space of two concentric cylinder molds with outer and inner diameters of 4 mm and 2 mm, respectively. Plain HRP-SF solutions were also injected within the cylindric molds and used as control conditions. The inner mold was a brass cylinder, whereas the outer mold was made of polypropylene. The system was placed in the sonicator at 37 °C for 2 h, in order to induce hydrogelation, to keep the homogeneous distribution of the ZnSr-β-TCP powders within the hydrogel matrix, and to increase the β-sheet content of the protein. After that, an immersion of approximately 15 sec in liquid nitrogen ensured the removal of the outer mold. Then, the inner mold was removed after immersion in ethanol 100% (*v*/*v*) for 1 h, also inducing a permanent β-sheet conformation to the tubes. The obtained tubes were then frozen at −80 °C overnight and freeze-dried (Telstar Cryodos-80, Barcelona, Spain) for at least 5 days. The HRP-SF/ZnSr-β-TCP composite tubular grafts are denominated as CTGs, and the plain HRP-SF tubular grafts are abbreviated as PTGs (Figure 1).

### 2.3. Physicochemical Characterization

#### 2.3.1. Scanning Electron Microscopy (SEM) and Energy Dispersive Spectroscopy (EDS) Analysis 

The macro-/micro-structures of the TGs were examined by SEM using a JEOL JSM-6010LV instrument (Tokyo, Japan; 10 kV and 16 mm working distance). Prior to analysis, the samples were sputter-coated with gold (Leica EM ACE600 coater; Leica Microsystems, Wien, Austria) for 60 s at 30 mA, 0.1 mbar and 50 mm working distance. The elemental composition of the scaffolds was determined by EDS using single-point analysis in three independent regions of the CTGs and PTGs (Pegasus X4M, Edax, Mahwah, NJ, USA; 10 kV and 11 mm working distance). 

#### 2.3.2. Micro-Computed Tomography (Micro-CT)

The micro-structure evaluation and 3D reconstruction of the TGs was performed using a high-resolution Micro-CT Skyscan 1072 scanner (Skyscan, Kontich, Belgium), with a pixel size of 15 µm. A standardized cone-beam reconstruction software (NRecon v1.4.3, SkyScan) was used for dataset reconstruction. A representative dataset of the slices was segmented into binary images with a dynamic threshold of 22–40 (gray values). Then, the images were used for the morphometric analysis (CT Analyser, v1.5.1.5, SkyScan, Edinburgh, UK), and to reconstruct the 3D models of the tubular structures (ANT 3D creator, v2.4, SkyScan). 

#### 2.3.3. Fourier Transform Infrared (FTIR) Spectroscopy

The chemical composition and conformation of the TGs were analyzed by FTIR spectroscopy (IRPrestige-21, Shimadzu, Kyoto, Japan) under an attenuated total reflectance (ATR) model (IRPrestige-21, Shimadzu, Kyoto, Japan). All spectra were obtained between 4600 cm^−1^ and 800 cm^−1^, at a 4 cm^−1^ resolution and an average of 50 scans. Three samples were used for CTGs and PTGs, respectively.

#### 2.3.4. X-ray Diffraction (XRD)

The crystallinity of the TGs was determined by XRD using a high-resolution Bragg-Brentano diffractometer (Bruker D8 Advance DaVinci, Karlsruhe, Germany) equipped with Cu Kα radiation (λ = 1.506 Å), produced at 40 kV and 40 mA. Data were collected in the 2θ range of 5–70° with a step size of 0.04° and 1 s per step. The analysis of the CTGs and PTGs was repeated three times, independently. 

#### 2.3.5. Mechanical Properties 

The mechanical properties of the TGs were evaluated under wet conditions using a universal mechanical testing machine (Instron model5540, Norwood, MA, USA), according to the ASTMC749–08 standard method. Before analysis, samples were hydrated in PBS solution until equilibrium was reached (overnight incubation). Then, the extremities of each sample were fixed by a grip and a load cell of 1 kN was used for measurements. A cross-head speed of 2 mm min^−1^ was employed and the samples were tested until rupture. Strain–stress curves were obtained from each experiment. The tensile Young’s modulus was determined using the initial linear region of stress–strain curves, by the tangent method. Five samples with 3 cm height were used per CTGs and PTGs.

The suturability of the TGs was assessed using a 4-0 suture placed 2 mm from the end of the CTGs and PTGs sutured to an ACL graft obtained from the right knee of a female pig, based on the long axis and strain to the rupture point of the samples. 

### 2.4. Swelling Ratio and Degradation Profile

The swelling ratio of the TGs was tested in PBS solution. The initial dry weight (*m_i_*) of samples (*n* = 4) was determined, followed by samples’ immersion in 5 mL of PBS solution. The analysis was performed at 37 °C incubation for a time period of 1 h up to 21 days. PBS solutions were renewed every 2 days. After each time-point, samples were blotted in filter paper to remove the excess of liquid, and the wet weight (*m_w_*) measured. The swelling ratio was determined using the following equation:

(1) $$\mathrm{Swelling}\;\mathrm{ratio}\;\left(\%\right)=\left[\frac{\left(m_{w}-m_{i}\right)}{\left(m_{i}\right)}\right]\times 100\%$$

The stability of the TGs was evaluated by performing an enzymatic degradation test, in which Protease XIV derived from (Streptomyces griseus, 3.5 U/mg) was dissolved in sodium azide solution (0.2 g/L) at 2 U/mL e 0.2 U/mL. The initial dry weight (*m_i_*) of CTGs and PTGs samples (*n* = 5) was measured, followed by samples’ immersion in 5 mL of protease solution. The analysis was performed at 37 °C incubation for a time period of 1 h to 21 days. The enzyme solutions were changed every 24 h. The dry weight (*m_f_*) of samples was determined after rinsing in distilled water and drying at 70 °C overnight. The weight loss of samples was determined using the following equation:

(2) $$\mathrm{Weigh}\;\mathrm{loss}\;\left(\%\right)=\left[\frac{\left(m_{i}-m_{f}\right)}{\left(m_{i}\right)}\right]\times 100\%$$

### 2.5. Bioactivity Assay in Simulated Body Fluid (SBF)

The in vitro bioactivity of the CTGs and PTGs was evaluated in SBF solution for 7 and 15 days (*n* = 3). The SBF solution was prepared as previously described [43], containing the same ions at similar concentrations as human blood plasma. TGs were immersed in SBF solution and incubated at 37 °C with continuous shacking (60 rpm). After each time-point, samples were rinsed in distilled water and allowed to dry at 37 °C for 2 days. The formation of apatite-like crystals on samples was then evaluated using SEM/EDS, following the procedure and parameters described in Section 2.3.1.

### 2.6. In Vitro Cell Studies

#### 2.6.1. SaOs-2 Cell Culture and Expansion

A human primary osteogenic sarcoma cell line (SaOs-2, ATCC, Manassas, VA, USA) was used to assess cell behavior in the TGs. Cells were cultured as monolayer in a standard Dulbecco’s modified Eagle’s medium (DMEM) supplemented with 10% (*v*/*v*) Fetal Bovine Serum (FBS; Biochrom, Merck, NJ, USA) and 1% (*v*/*v*) of antibiotic-antimycotic mixture (Life Technologies, Carlsbad, CA, USA), at 37 °C and 5% CO_2_ incubator. Cells were cultured until confluence by exchanging the culture medium every 2–3 days. 

#### 2.6.2. Seeding of SaOs-2 in the TGs 

For cell seeding, longitudinal cuts were performed to TGs of 10 mm of length and used in halves. Cells were seeded at a density of 3 × 10^5^ cells per 5 µL cell suspension in the internal surface of the pre-wet CTGs and PTGs. The constructs were kept for 3 h under incubation and then completed with 1 mL of standard culture medium. Samples were cultured under static culture conditions and harvested after 1, 7 and 14 days. Culture medium was renewed every 2–3 days.

#### 2.6.3. Live/Dead Staining

Cell viability within the TG constructs was observed by performing a calcein-AM (1 µg/mL) and propidium iodide (PI: 5 µg/mL) staining. Briefly, three samples of each group (CTGs and PTGs) were washed in PBS solution and incubated in the dark for 30 min, at 37 °C and 5% CO_2_ incubator in calcein-AM and PI solutions. Before analysis, samples were washed again in PBS solution to remove excessive background and immediately observed under fluorescence microscopy in a laser confocal microscope (Leica TCS SP8; Leica, Wetzlar, Germany).

#### 2.6.4. Alamar Blue Assay

The metabolic activity of cells in the CTG and PTG constructs was quantified using an AlamarBlue^®^ (BioRad, Hercules, CA, USA) assay. After each time point, a 10% (*v*/*v*) Alamar blue solution was prepared in standard culture medium and added to samples placed in different culture wells. The system was incubated in the dark for 4 h at 37 °C and 5% CO_2_ incubator, followed by fluorescence monitoring at 530 nm/25 nm excitation and 590/35 nm emission using a microplate reader (FL 600, Bio-Tek Instruments, BioTek, Winooski, VT, USA). Samples were carefully washed in PBS solution and kept in fresh culture medium until further analysis. Three samples of each group (CTGs and PTGs) were tested at each time-point, in three independent experiments. TGs without cells were used as control.

#### 2.6.5. SEM Analysis

Cell adhesion and distribution within the CTGs and PTGs were observed by SEM. After each time-point, the constructs were washed with PBS solution and fixed for 1 h at 4 °C in a 2.5% (*v*/*v*) glutaraldehyde solution. Then, samples were dehydrated in a series of ethanol concentrations (30 to 100% *v*/*v*) and left to dry overnight. Prior to the SEM observations, all constructs were sputter-coated with gold (Section 2.3.1).

#### 2.6.6. dsDNA Quantification

Cell proliferation was determined using a fluorimetric double-strained DNA (dsDNA) quantification kit (Quant-IT PicoGreen dsDNA Assay Kit 2000 assays; Life Technologies, Carlsbad, CA, USA), according to manufacturer’s instructions. Briefly, after each time-point the cell-seeded CTGs and PTGs samples were washed in PBS solution, and frozen at −80 °C in 1 mL of ultrapure water. For the DNA quantification, TG constructs were defrosted and ultrasonicated for 1 h to complete the release of DNA from samples. Fluorescence monitoring was performed at 485/20 nm excitation and 528/20 nm emission using a microplate reader. A standard curve for DNA concentration analysis was prepared with concentrations ranging from 0 to 2 µg/mL. Three samples of each group (CTGs and PTGs) were tested at each time-point, in three independent experiments. TGs without cells were used as control.

#### 2.6.7. Alkaline Phosphatase (ALP) Quantification

The amount of ALP in samples was quantified by using an ALP quantification kit, applied to the same samples collected for dsDNA quantification (Section 2.6.6), after lysis and freezing at −80 °C in ultrapure water. For the ALP quantification, 20 µL of Alkaline Buffer Solution at 1.5 M, 100 µL of Alkaline Substrate Solution (1 capsule diluted in 25 mL of ultrapure water), and 80 µL of sample were added to a well of 96-well white polystyrene plate and incubated for 1 h at 37 °C in the dark. In the meantime, standard solutions were prepared with concentrations ranging from 0 to 250 µM using a p-nitrophenol solution (1 mM). After incubation, 100 µL of NaOH solution (0.3 M) were added to stop reactions (samples and standards) and the absorbance was measured at 405 nm using a microplate reader. Three samples of each group (CTGs and PTGs) were tested at each time-point, in three independent experiments. TGs without cells were used as control.

#### 2.6.8. Alizarin Red Staining

The matrix mineralization at the surface of the CTGs and PTGs constructs was assessed by performing an alizarin red staining. First, the samples were washed with PBS solution and fixed in 10% (*v*/*v*) formalin for 1 h at room temperature (RT). A 2% (*w*/*v*) Alizarin Red S sodium salt (Thermo Fisher Scientific, Waltham, MA, USA) solution prepared in ultrapure water was used for TGs staining for 5 min. Samples were repeatedly washed until all the background solution was removed, and observed under a stereomicroscope (Model Stemi 2000-C; Zeiss, Jena, Germany) coupled with the Zen 3.2 digital image processing software (Blue Edition; Zeiss, Jena, Germany).

#### 2.6.9. RNA Isolation and Real-Time Quantitative Reverse Transcriptase-Polymerase Chain Reaction (RT-PCR)

The mRNA expression of osteogenic genes of interest was determined by real-time RT-PCR analysis. For that, the total mRNA from the CTGs and PTGs constructs was first extracted using the Direct-zol^TM^ RNA MiniPrep kit (Zymo Research, Irvine, CA, USA), according to manufacturer’s instructions. Briefly, the TGs constructs were washed in PBS solution and kept in 300 µL of TRI Reagent^®^ at −80 °C, until further use. Samples were then defrosted at RT and sonicated for 1 h to ensure complete lysis and removal of cells from the scaffolds. RNA quantification and purity were assessed by Nano-Drop ND-1000 spectrophotometer (Nano-Drop Technologies, Wilmington, DE, USA). Complementary DNA (cDNA) synthesis was performed by using 200 ng of the total RNA extracted from samples by random priming with qScript Reverse Transcriptase (RT). The obtained single-stranded cDNA of each sample was then used as template for the amplification of the genes of interest (primers in Table 1) using the PerfeCta SYBR Green FastMix kit (Quanta Biosciences, Gaithersberg, MD, USA). The transcripts expression data were normalized through the endogenous housekeeping gene glyceraldehyde 3-phosphate dehydrogenase (GADPH), and the relative gene expression was determined using as a calibrator the expression levels of SaOs-2 cells collected at the time of cell seeding (day 0), according to the Livak (2^−ΔΔCT^) method. Three samples of each group (CTGs and PTGs) were tested at each time-point, in three independent experiments. 

### 2.7. Statistical Analysis

All numerical results were presented as mean ± standard deviation (SD). Statistical analysis was performed using the GraphPad Prism 7.0 software (GraphPad Software, La Jolla, CA, USA). A Shapiro–Wilk normality test was first applied to establish about data normality. The results obtained from tensile modulus were evaluated by means of a parametric unpaired *t*-test. For biological assays, a non-parametric Kruskal–Wallis analysis followed by Dunn’s multiple comparison test was performed to compare the differences between more than two groups, whereas the non-parametric Mann–Whitney test was applied to compare the differences between two independent groups. The significance levels were set to * *p* < 0.05, ** *p* < 0.01, *** *p* < 0.001, **** *p* < 0.0001.

## 3. Results and Discussion

In the field of TE, tubulization approaches have been mostly applied as soft tissue substitutes, including vessels [44], or nerve conduits [31], being capable of mimicking important biological and structural aspects of such tissues. TGs have also been applied as a valuable TE strategy for ACL reconstruction, being used as ACL substitutes in surgical methodologies [15]. However, the application of TGs as bone fillers has been recognized as a potential solution for the recurrent post-surgical bone tunnel enlargement [45]. The strategy herein presented takes advantage of the well-recognized structural and mechanical properties of SF, combined with ion-doped β-TCP powders and enzymatically cross-linked through the HRP/H_2_O_2_ complex, which allowed to obtain strong but yet resilient structures with adequate features for bone tunnel defects filling. In fact, these hydrogels represent an innovative application of SF that had never been explored before as fiber-based grafts for ACL substitution and reconstruction. Using the HRP-mediated cross-linking, more stable and adjustable 3D composite networks were produced [31], together with superior crystallinity and mechanical properties induced by the presence of the inorganic materials [27,28,29]. Thus, the proposed CTGs were produced by mixing the HRP-cross-linked SF solution with the ZnSr-β-TCP particles before hydrogelation. Afterwards, the cylinder molds were subjected to a sonication process in order to stabilize and uniformize the ZnSr-β-TCP particles distribution within the HRP-SF. Through this system, it was possible to complete faster the hydrogelation at 37 °C, while the β-sheet structure was incremented. Previously, a similar system was proposed to produce tubular conduits (TCs), however, the hydrogelation process was completed at 37 °C without sonication [31]. By this means, the newly formed TCs achieved an amorphous conformation reversed to a permanent β-sheet only after immersion in ethanol. After removing the molds, the obtained hollow tubes were freeze-dried to be kept in stable conditions until further characterization. The PTGs were produced under the same conditions but without ZnSr-β-TCP incorporation to be used as control (Figure 1). Therefore, the effects of the ZnSr-β-TCP on TGs morphology, crystallinity, mechanical properties and biological performance were explored.

### 3.1. Morphology and Macro/Microstructure Characterization

**Figure 2:**
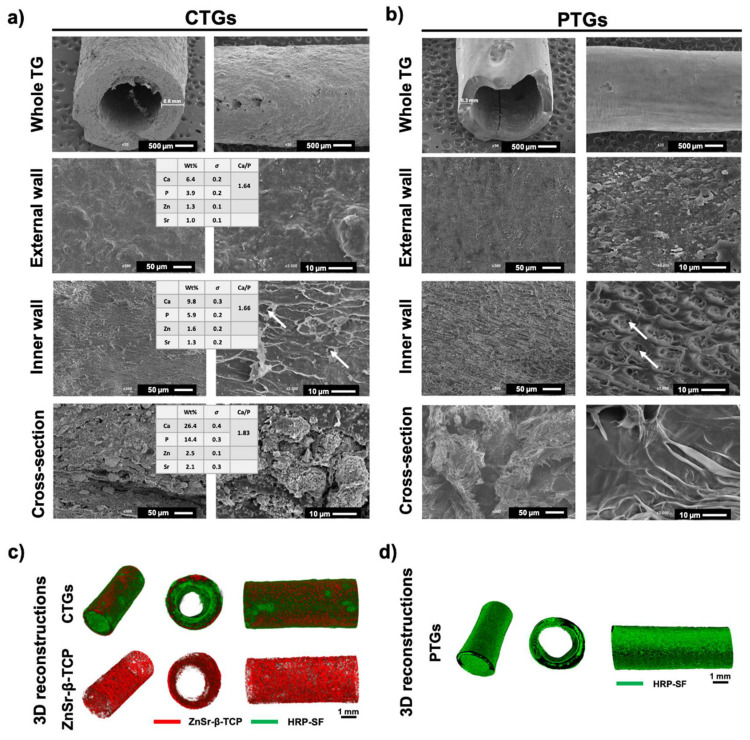
Macro/microstructure of the developed TGs. Representative SEM micrographs of the (**a**) CTGs and (**b**) PTGs, and EDS elemental analysis with the detected ion elements (Zn and Sr) and Ca/P ratios determined in the different regions of the CTGs (external, inner and cross-section regions). The white arrows indicate micropores (<10 μm). Wt% stands for percentage by weight and σ stands for standard deviation. Micro-CT reconstructions of the (**c**) CTGs and (**d**) PTGs, exhibiting the ZnSr-β-TCP component distribution in the TGs (in red) and the HRP-SF hydrogel matrix (in green).

The morphology and macro/microstructure of the CTGs and PTGs were assessed by SEM and micro-CT (Figure 2). It was observed that the CTGs presented higher wall thickness of ≈0.6 mm as compared to the half wall thickness of ≈0.3 mm presented by the PTGs (Figure 2a). The differences in wall thickness can be related to the ZnSr-β-TCP component retained in the hybrid TGs. It has been reported that the crystalline phase of SF can nucleate the deposition of HAp nanoparticles [46], as well as the amorphous regions of the protein can be blended with Hap, inducing conformational changes (higher β-sheet content) and affecting the crystallinity, morphology and mechanical properties of the SF structures [26]. In Figure 2a,b, the microstructure of the CTGs and PTGs showed evident differences in terms of external, inner and cross-section walls’ morphology. The external surface of the CTGs presented porosity at nanoscale (Figure 2a), whereas some microporosity (≈3–6 μm) was also observed in the inner wall. Moreover, the presence of the ZnSr-β-TCP powders more concentrated in the internal structure of the CTGs (cross-sections) is evident. From the 3D reconstructions of the CTGs (Figure 2c), it was possible to confirm the presence and uniform distribution of the ZnSr-β-TCP component within the HRP-SF hydrogel matrix. Moreover, the EDS spectra analysis revealed the presence of calcium (Ca) and phosphorous (P) ions in the external (Ca: 6.4 ± 0.2%; P: 3.9 ± 0.2%) and inner (Ca: 9.8 ± 0.3%; P: 5.9 ± 0.2%) surfaces of the CTGs, with Ca/P ratios of 1.64 and 1.66, respectively. These values were similar to those typically found for the HAp (1.67) and adult human bone (1.71) [47,48]. In the cross-section region, higher intensity of the Ca (26.4 ± 0.4%) an P (14.4 ± 0.3%) peaks were detected, reaching a Ca/P ratio of 1.83. As expected, Zn^2+^ (External wall: 1.3 ± 0.1%; Inner wall: 1.3 ± 0.2%) and Sr^2+^ (External wall: 1.0 ± 0.1%; Inner wall: 1.3 ± 0.2%) were also detected at the surface of the CTGs, reaching higher values in the cross-section region (Zn: 2.5 ± 0.1%; Sr: 2.1 ± 0.3%). Regarding the PTGs (Figure 2b), an organized microporosity was observed at the inner surface of the TGs, mainly with two types of pore sizes. Micropores of ≈10 μm were observed, as well as micropores with less than ≈2 μm distributed inside the micropore walls. The SEM images of the PTGs cross-sections reveal a high degree of porosity. However, no traces of ZnSr-β-TCP were detected, as confirmed by the 3D reconstructions of the control PTGs (Figure 2d). The level of porosity and incorporation of CaPs are important aspects for the success of the CTGs in promoting efficient osteogenesis, while filling the bone tunnel space in ACL reconstruction [27,40,41]. Small pore sizes (<50 μm) are recommended to promote cell adhesion, nutrient diffusion and scaffold colonization for a faster mineralization and osteointegration [26]. In this case, larger and interconnected pores should be avoided in order to minimize the diffusion of cells and newly formed bone ECM into the TG lumen and ligament graft microenvironment preventing the formation of fibrous tissue, which affects efficient tissue regeneration [49]. Although SF has poor natural capabilities to promote bone regeneration, it is well established that its blending with CaPs is advantageous for improving scaffold degradability, mechanical performance, and osteogenic capacity [50,51]. Previous studies have showed that polymeric matrices combined with β-TCP presented higher tendon-to-bone osteointegration in a ACL reconstruction [15]. For example, SF fiber-based grafts combined with TCP [22] and HAp [23] were proposed for ACL repair showing osteoconductive capacity during bone ingrowth.

### 3.2. Chemical Structure and Mechanical Properties

**Figure 3:**
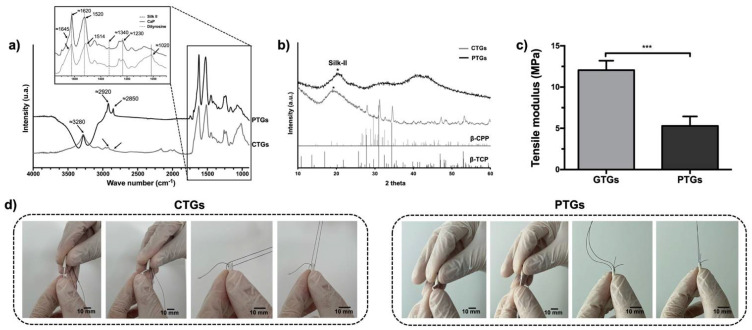
Structural composition and mechanical characterization. (**a**) ATR-FTIR spectra and (**b**) XRD patterns, showing the chemical structure of SF on the CTGs and PTGs, and the characteristic peaks of β-TCP and β-CPP belonging to the ZnSr-doped β-TCP powders. (**c**) Tensile modulus of the CTGs and PTGs determined from the respective stress–strain curves (Figure S1). *** *p* < 0.005 (**d**) Representative suturability tests performed on the CTGs and PTGs by wrapping a pig knee ACLG.

The SF chemical structure and crystallinity were assessed by ATR-FTIR (Figure 3a) and XRD (Figure 3b). The FTIR spectra showed a characteristic peak of SF at ≈3280 cm^−1^ for both CTGs and PTGs, specific to the N-H (amide A) and O-H stretching bonds. The peak associated to amide B (≈3070 cm^−1^) was barely pronounced on the CTGs and presented as a shoulder peak for the PTGs. The peaks at ≈2920 cm^−1^ and ≈2850 can be assigned to C-H asymmetric stretching from methyl groups [52]. The intensity of these peaks is higher for the PTGs, which can be related to the contribution of the ZnSr-β-TCP powders incorporated into the SF-based hydrogel structure. The next FTIR region (enlarged area of graph in Figure 3a) is relative to the amide I, II, and III bands, showing strong absorbance peaks at ≈1620 cm^−1^ for both CTGs and PTGs. These can be assigned to amide-I band (C=O stretching from the backbone) and are indicative of β-sheet conformation [26,32,37]. The second highly pronounced absorbance peak at 1520 cm^−1^ for the PTGs can be assigned for amide-II band (N-H bending and C-H stretching) and is also indicative of the β-sheet conformation on SF [37,53]. As for the CTGs, a small shift of the amide-II signature band was observed towards a lower wave number (1514 cm^−1^), which indicates higher β-sheet content in these structures [32]. This result suggest that the blending with ZnSr-β-TCP increased the crystalline β-sheet state of SF. Confirming this, the CTGs presented a distinct absorbance peak at ≈1020 cm^−1^ characteristic of the vibration from the phosphate (PO_4_^3−^) in the CaP [38,39]. Furthermore, the appearance of an additional absorbance peak at ≈1230 cm^−1^, correspondent to the amide-III band can be assigned to C-N stretching and N-H bending and it can also be attributed to the β-sheet conformation of SF on both CTGs and PTGs [31,52,53,54]. Nevertheless, hardly pronounced shoulder peaks were observed at ≈1645 cm^−1^ corresponding to the contribution of the random coil conformations in the structure of the SF hydrogels, confirming previous reports regarding HRP-cross-linked SF structures [26]. Moreover, the appearance of intensity of bands at ≈1340 cm^−1^ for both CTGs and PTGs assigned to the stretching vibration of the dityrosine C-C bonds [53], also confirms the HRP-mediated cross-linking reaction on SF hydrogel structures formation. XRD patterns of the CTGs and PTGs are also displayed in Figure 3b. The most typical crystalline XRD peak assigned for SF appears at 20.8° [38,39]. This peak was highly pronounced on the PTGs with a crystallite size of 34.0 Å, confirming the β-sheet structure on the plain SF. The incorporation of the ZnSr-β-TCP powders resulted on a small peak shift on the CTGs towards lower 2θ ≈ 20° and in a decrease of the SF crystallite size to 27.8 Å, confirming the higher silk II structure on the CTGs [42,55]. Additionally, the typical crystalline peaks belonging to β-TCP (standard ICDD PDF 04-014-2292) and trace amounts of β-calcium pyrophosphate (β-CPP) (standard ICDD PDF 04-009-3876) in the CTGs composites were observed [27,42]. Figure 3c displays the mechanical properties of the CTGs and PTGs. The mechanical performance of the composites is extremely important regarding a successful ACL grafts implantation [56]. In fact, the mechanical behavior of the ACLGs upon implantation is not only dependent on the ACLG properties, but also on the graft fixation technique coupled with the bone tunnel [10]. As an example, the hamstring tendon and bone-patellar tendon-bone, which are the most popular autografts used for ACL replacement, require interference screws (IFS) for fixation into the tunnels [57]. Suture post-screws are also used for grafts fixation outside the tunnels, as it is the case of the Endobutton technique [58]. All these features influence the initial tensile load and mechanical strains of the grafts. Herein, the stress–strain curves of the CTGs and PTGs (Figure S1) revealed that the tensile modulus of the hydrated CTGs was significantly higher (12.05 ± 1.03 MPa) than that of the PTGs (5.30 ± 0.93 MPa). These are expected results, considering that the presence of the inorganic component on the composites affected the silk II crystalline structure of SF (higher β-sheet), confirmed by FTIR and XRD analysis (Figure 3a,b) [22,23]. Moreover, it also reflected the higher ability of the CTGs to support the mechanical changes in length when subjected to longitudinal tension, which is important, considering that the bone tunnel fillers wrapping the ACLGs must attain some rigidity while they elongate under strong tensile loads. Previous studies reported that the tensile modulus of native ACL tissue, considering male and female samples, ranged between 128 ± 35 MPa and 99 ± 50 MPa, respectively [59]. These values are not in the same magnitude to that obtained for the CTGs. Nevertheless, it is important to consider that the proposed CTGs are not expected to substitute the mechanical properties of the native ACL, or even ACLGs, but to support and fill the gap between the grafts and the bone tissue. For instance, Li et al. [22] showed that the tensile strength of a TCP block (≈500 N) to anchor a silk-based ACL graft into the bone tissue was lower than that of the silk graft (≈900 N) used as an ACL substitute. Moreover, in bone tissue regeneration, the mechanical properties of the temporary tissue substitutes are not expected to equal those of the native bone tissue. Scaffolds must provide sufficient mechanical support to allow cells to recognize the mechanical stimuli necessary for new ECM formation, mineralization, and ultimately tissue regeneration [60]. 

Finally, when TGs are considered to substitute or to connect to the native tissues in surgical approaches, the suturability properties of those grafts are of extreme importance [31]. In the particular case of the CTGs used as bone tunnel fillers, they must hold structural stability and integrity to be saturable and resistant to surgical manipulation and to graft fixation techniques. The suturability tests showed that an ACL graft obtained from a female pig knee was able to be sutured to the CTGs and PTGs by wrapping the end of the grafts. The sutures showed to be resistant when pulled in and out, which simulates the mechanical strength suffered during grafts implantation and fixation (Figure 3d). Moreover, no differences were noticed when comparing the breaking strength of the CTGs (Video S1) and PTGs (Video S2) after suturing, which indicates that the incorporation of the ZnSr-β-TCP powders did not affect the toughness of the HRP-SF hydrogel matrix.

### 3.3. Swelling Ratio, Degradation Profile and Bioactivity Evaluation

**Figure 4:**
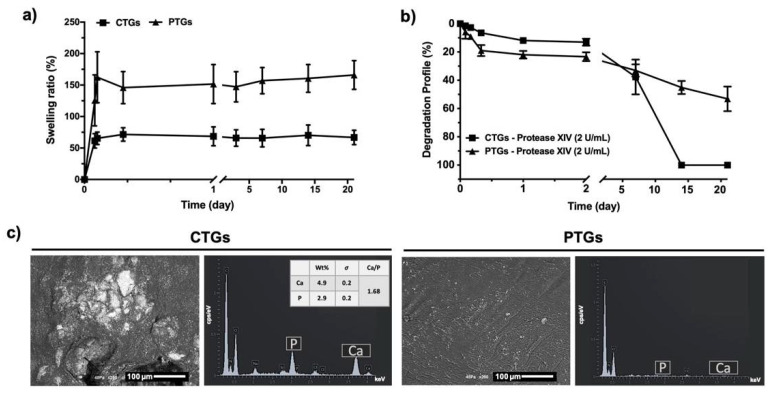
Swelling ratio, degradation profile and in vitro bioactivity. (**a**) Water uptake profile and (**b**) weight loss of the CTGs and PTGs immersed in PBS solution and protease XIV (2 U/mL) solution, respectively, for a period of 21 days (wt.%). (**c**) Representative SEM micrographs and EDS elemental analysis of the CTGs and PTGs after 7 days of immersion in SBF solution. σ stands for standard deviation.

The swelling properties of the CTGs and PTGs are important parameters of TGs proposed as bone fillers in ACL replacement. In fact, scaffolds must fulfill the gap between the ACLG and the bone tissue post-implantation, which implies swelling capabilities for adaptation to the surrounding tissues. As previously mentioned, ACL graft fixation techniques may lead to bone tunnel enlargement, which implies the existence of bone tunnel fillers attached to the ACLGs, ensuring the accurate osteointegration of the selected grafts. Hence, scaffolds with swelling capabilities are highly recommended [10]. Figure 4a shows that both structures presented swelling capacity, reaching an equilibrium after 4 h of immersion in PBS solution (CTGs: 65.47 ± 9.9 wt.%; PTGs: 162.4 ± 40.5 wt.%) and maintaining their weight up to 21 days. The stability of the swelling properties of the TGs is important considering that the scaffolds themselves can induce tunnel augmentation, and the occurrence of a swelling/deswelling behavior would affect ACLGs mechanical response and osteointegration [22,61]. Moreover, the CTGs presented a lower swelling capacity as compared to the PTGs, which can be justified by the incorporation of the inorganic component that also increased the β-sheet content and crystallinity of SF (Figure 3a,b). This molecular conformation of the protein makes it more hydrophobic due to the highly ordered and tight polymeric structure, which ultimately affects the hydration capacity of the hydrogel-based TGs [62]. Previously, Yan et al. [38] also showed that SF/nano-sized CaP scaffolds presented stable and lower hydration capacity than that observed on pure SF scaffolds.

The degradation profile of the CTGs and PTGs was analyzed under protease XIV solution (Figure 4b) [26,27]. This enzyme has been extensively reported for silk degradation studies, promoting a non-specific proteolytic activity towards the chemical structure and amino acids of SF [26,27,31]. Thus, it is expected that SF demonstrates a higher degradation in response to proteinase XIV, or simulates the synergistic effect of several enzymes with specific activity on the amino acid sequence of the protein (i.e., chymotrypsin, collagenase, metalloproteinases) [63,64]. It was observed that the weight loss of the CTGs and PTGs was more stable under low concentration of protease XIV (0.2 U/mL, Figure S2). The degradation profile observed for the CTGs (13.1 ± 2.3 wt.%) was slightly slower than that observed for the PTGs (23.3 ± 3.0 wt.%) up to 2 days of incubation with protease XIV (2 U/mL). However, both types of TGs started to lose integrity between day 2 and day 7, presenting a weight loss of 37. 7 ± 12.3 wt.% on the CTGs and 33.1 ± 4.2 wt.% on the PTGs. From day 7, a constant decrease in weight loss of the PTGs was observed reaching half of their weight (53.2 ± 8.6 wt.%) after 21 days of incubation. This behavior was not observed for the CTGs, which were completely degraded after 14 days in proteolytic solution (2 U/mL). These differences can be attributed to the incorporation of ZnSr-β-TCP into the SF structure under cross-linking, which, at an early stage, hindered the proteolytic cleavage induced by the degradation enzyme [27], but started to be dissolved from the scaffolds’ matrix over time, making them more unstable and prone to proteolytic degradation [39]. Previous studies also reported that the higher weight loss of SF/nano-CaP scaffolds as compared to the control SF scaffolds could be attributed to the partial dissolution of the crystalline CaPs observed in the degradation solution [38,39]. Nevertheless, the authors also stated that the release of Ca and P from the SF/nano-CaP scaffolds could be an advantage to promote faster bone regeneration [39].

The in vitro bioactivity tests performed to evaluate the biomineralization potential of the TGs showed that only the CTGs induced the formation of apatite-like crystals after 7 days (Figure 4c) and 15 days (Figure S2) of immersion in SBF solution, owing the presence of ZnSr-β-TCP on the composites CTGs. In a study performed by Carvalho et al. [31], no apatite-like crystals were observed on the surface of freeze-dried conduits even after 30 days of immersion of HRP-SF-based TCs in SBF solution. However, the apatite-forming capacity was dependent on the conduits’ surface microstructure and mechanical properties, which varied according to the final processing method (i.e., freeze-drying, drying or no drying). Herein, the superior mechanical properties of the CTGs may have a positive influence on the scaffold’s bioactivity. Indeed, the mutual influence of scaffold stiffness and biomineralization capacity has been previously reported [65,66]. This is crucial for the success of the developed CTGs as bone tunnel fillers in ACL regeneration approaches, suggesting that these composites can be integrated into the bone tunnel space and promote faster osteointegration and bone regeneration. EDS analysis confirmed the formation of a mineralized matrix with the presence of Ca and P elements, with a Ca/P ratio of 1.68, in the range of the Ca/P values typically obtained for HAp (1.67) in an adult human bone (1.71) [47].

### 3.4. Cell Viability, Proliferation and Morphological Profile

**Figure 5:**
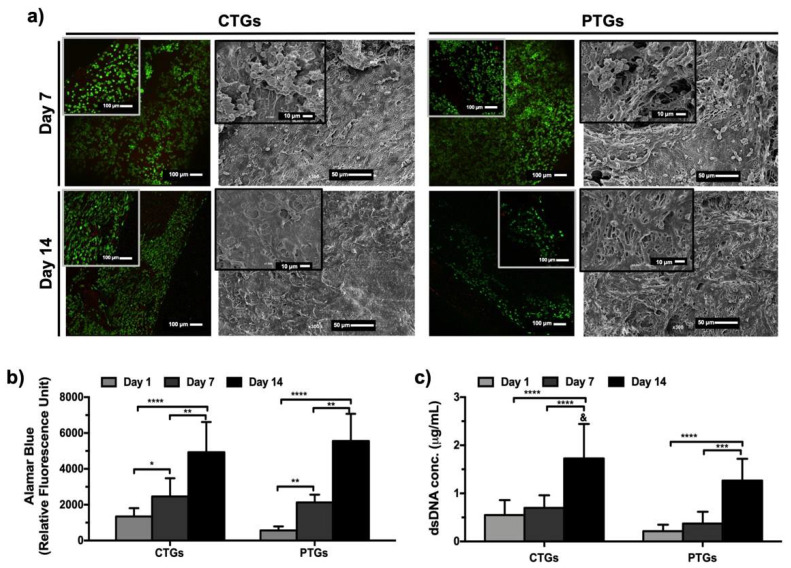
In vitro cell characterization. (**a**) Live/dead staining and SEM micrographs showing cell viability and morphology on the CTGs and PTG, after 7 and 14 days of culture. Living cells are stained in green and dead cells are stained in red. (**b**) Alamar blue assay detecting metabolic activity of cells, and (**c**) dsDNA quantification to analyze cell proliferation on the CTGs and PTGs, up to 14 days of culture. ^&^ Statistically significant when compared to the PTGs at the same culture period (* *p* < 0.05; ** *p* < 0.01; *** *p* < 0.005; **** *p* < 0.0001).

As previously discussed, the developed CTGs are expected to behave as bone tunnel fillers in ACLGs implantation scenarios promoting grafts osteointegration and simultaneously bone tissue regeneration [21]. Thus, SaOs-2 cells were used to demonstrate the potential of the CTGs as matrices for osteogenic cells activity, proliferation, and bone matrix formation/mineralization. This is the first step to further validate the composites for osteogenic differentiation strategies in long-term cultures, or as an interface capable of simultaneously promote osteogenic and ligament activity on the different sides of the tubular structure. Figure 5a shows the results obtained from live/dead staining, where it was found that cells adhered and remained alive up to 14 days of culture, on both CTGs and PTGs. A high cell density was observed at day 7, and at day 14, the cells showed an elongated morphology and stretched around the tubular structures, as confirmed by SEM micrographs. Moreover, from SEM images, a slightly distinct morphological pattern was observed at day 14 on the CTGs constructs, with cells completely elongated and covering the scaffolds surface with extended lamellipodia. This is an expected cell behavior and typically observed on SaOs-2 cells cultured with CaP-based substrates with desirable properties for osteogenesis and bone tissue regeneration [67]. Alamar blue assay (Figure 5b) confirmed cell viability results with a significant increase of cellular metabolic activity up to 14 days of culture on the CTGs and PTGs. No differences were registered when comparing both structures, indicating that the different surface morphologies of the CTGs and PTGs (Figure 2a,b) were not impactful enough to induce different viability and metabolic activities on cells. Nevertheless, the results obtained from dsDNA quantification showed not only a significant increase of cell proliferation over the 14 days of culture on both structures, but also that at day 14, the DNA content was significantly higher on the CTGs, as compared to the PTGs (Figure 5c). These results are in good agreement with SEM observations (Figure 5a) and they may be an indication that the presence of the ZnSr-β-TCP on the TGs positively affected SaOs-2 cell proliferation and activity. Previously [67], SaOs-2 cells cultured in direct contact with nanosized HAp also showed a high metabolic activity, proliferation degree, and nanoparticles internalization ability. SF scaffolds modified or coated with HAp nanoparticles could stimulate the proliferation, differentiation, and osteogenesis of bone marrow mesenchymal stem cells (BMSCs), to be further applied as ligament grafts in bone-ligament defects repair and ACL reconstruction [23,68]. SF has been successfully applied for ACL TE approaches [20,25,69], however, the osteointegration ability of the SF-based scaffolds is usually ensured by incorporating inorganic components that subsequently accelerate bone tunnel regeneration [22].

### 3.5. Biochemical Characterization and ECM Mineralization

**Figure 6:**
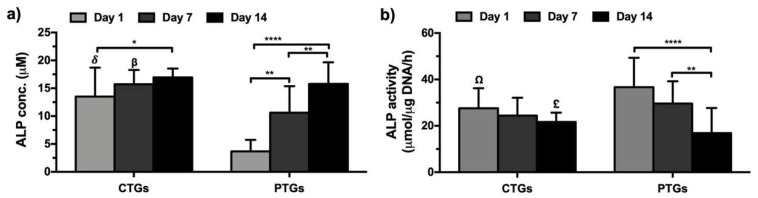
Quantification of in vitro osteogenesis and mineralization profile. (**a**) ALP quantification and (**b**) normalized ALP activity in the CTGs and PTGs cultured up to 14 days with SaOs-2 cells. ^δ,β,Ω,£^ Statistically significant when compared to the PTGs at the same culture period (Ω* *p* < 0.05; β,£** *p* < 0.01; δ**** *p* < 0.0001).

The ALP concentration of the SaOs-2 cells cultured up to 14 days on the CTGs presented at the early time-points (day 1 and day 7) significantly higher levels as compared to that observed on PTGs (Figure 6a). Nevertheless, the incorporated ZnSr-β-TCP powders seem to have a higher influence on ALP production in the early stages of culture, reaching at day 14 similar levels to that observed on the PTGs. These are expected results, considering the well-known influence of CaPs on the production and activity of this early marker of osteogenesis [27]. Moreover, the observed bioactive properties of the CTGs confirmed after the scaffolds’ immersion in SBF solution for 7 days (Figure 4c) also corroborate this hypothesis. The PTGs also showed prominent osteogenic properties by the significant increase of ALP levels along the culture period [60]. The ALP activity of cultured cells was normalized by the corresponding dsDNA values in the scaffolds (Figure 6b). Results showed that the ALP activity remained statistically similar over the 14 days of culture on the CTGs. However, when normalized to the dsDNA of cells, the ALP production on the PTGs significantly decreased from day 1 until day 14, presenting at day 14 significantly lower levels than that observed in the CTGs. These results can be explained by the lower DNA amount quantified on the PTGs (Figure 5c). ALP is a highly representative bone marker of osteoblasts, which hydrolyzes the phosphate ion for the formation and deposition of HAp in ECM mineralization [70]. Thus, the ALP activity patterns presented by the CTGs and PTGs can be related to the increase in ECM mineralization confirmed at day 14 from alizarin red staining (Figure S3), suggesting that both structures have potential for new bone deposition in ACL osteointegration approaches [60].

### 3.6. Osteogenic Genotype Evaluation

**Figure 7:**
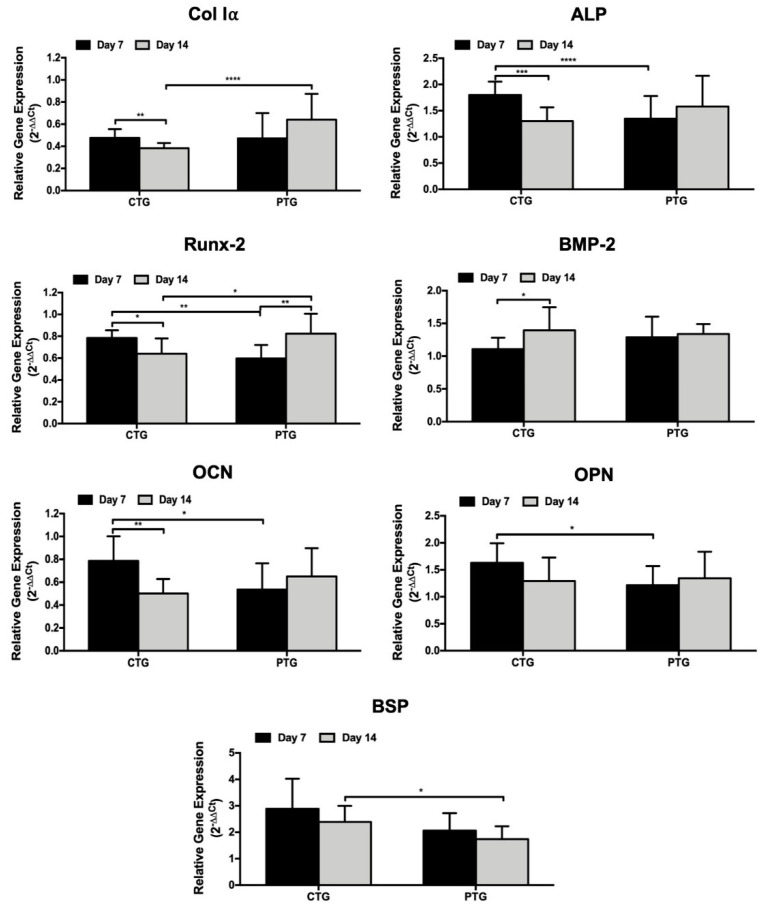
Gene expression profile of osteogenic-related markers. Real-time RT-PCR results of the osteogenic-related transcripts Col Iα, ALP, Runx-2, BMP-2, OCN, OPN and BSP by the SaOs-2 cells cultured in the CTGs and PTGs up to 14 days.(* *p* < 0.05; ** *p* < 0.01; *** *p* < 0.005; **** *p* < 0.0001).

The analysis of the gene expression profile, after 7 and 14 days of culture on the CTGs and PTGs, revealed that the SaOs-2 cells expressed the typical osteogenic-related markers Col Iα, ALP, BMP-2, Runx-2, OCN, OPN and BSP (Figure 7). The analysis showed that the expression levels of Col Iα were significantly higher at day 7 on the CTGs, as compared to day 14, as expected considering that Col Iα is an early marker of osteoblasts [71]. At the same time, the osteogenic potential of the incorporated ZnSr-β-TCP powders was evidenced by the late and significant up-regulation of Col Iα observed after 14 days of culture on the PTGs. ALP and Runx-2 markers showed a similar gene expression profile on both CTGs and PTGs to that observed for Col Iα. Considering that ALP and Runx-2 are early markers of osteogenic activity and are deeply involved in bone development [71,72], these results confirm the positive influence of the ZnSr-β-TCP on CTGs bioactivity and osteogenic performance. A previous study also reported the up-regulation of Col I and Runx-2 markers on knitted SF-based ACLGs modified at both ends with low crystallinity HAp [23], supporting the large potential of SF-CaP composites in ACLGs osteointegration. Regarding the gene expression of BMP-2, a significant upregulation was observed after 14 days of culture on the CTGs. Nevertheless, when compared the expression on the CTGs and PTGs, no significant differences were observed at the tested culture periods. Several studies demonstrated that BMP-2 can upregulate the expression of early and late osteogenic markers, including ALP, Runx-2 and OCN [73], being also responsible for accelerating ECM mineralization [74]. This is in good agreement with the results obtained for Ca deposition (Figure S3), in which the formation of a mineralized ECM was observed after 14 days of culture on the CTGs and PTGs. Interestingly, the results showed that the expression levels of OCN, OPN, and BSP were similar with higher tendency at day 7 than at day 14 on the CTGs. Considering that these are late markers of osteogenic activity [72], their early and significantly higher expression on CTGs, as compared to the PTGs, reinforces the osteogenic potential of ZnSr-β-TCP powders. 

As main future perspectives of this study, we expect that the incorporation of the micro-/nano-sized ZnSr-β-TCP particles will endow the HRP-SF-based TGs with properties of osteoconductivity capable of inducing osteogenic differentiation and mineralization in long-term cultures with mesenchymal stem cells. Moreover, further studies including the development of multifunctional CTGs for ligament-to-bone interface regeneration are planned. The dual surface functionalization of these CTGs might be helpful to simultaneously guide osteogenic regeneration and ligament cells activity. Combining the achievements of this study, we hope to enhance the drug delivery properties of grafts by loading the ZnSr-β-TCP particles before incorporation into the scaffolds, or by following other green chemistry methods and supercritical fluid processing to tune/load both the inner and external surfaces of the CTGs with desired biological factors. 

## 4. Conclusions

The present study proposed the development of HRP-crosslinked SF tubular scaffolds fully integrating ZnSr-β-TCP powders, to be used as bone tunnel fillers in anterior cruciate ligament grafts regeneration approaches. From a medical perspective, the developed CTGs are of extreme importance, as the occurrence of bone tunnel enlargement is deeply associated with the surgical processes and post-surgical recovery upon ACLGs implantation. Thus, the obtained structures are expected to fill the space between the implanted ACLGs and the bone tissue, promoting graft stabilization, osteointegration, and bone tissue regeneration. These composites presented adequate structural integrity, swelling capacity, and tensile strength for ACLGs fixation. The obtained tensile strength of the CTGs is of capital importance, justified by the homogeneous ZnSr-β-TCP component distribution alongside with the tubular structures. CTGs showed the ability to support SaOs-2 cells adhesion, viability, and proliferation up to 14 days of in vitro culture. The osteogenic activity of the CTGs was represented by the superior ALP production and genotypic expression of osteogenic-related markers, allied to the formation of a mineralized matrix. Although complementary in vitro and in vivo studies are necessary to validate the CTGs osteogenic differentiation and capable of simultaneously promote bone formation and ligament tissue activity on the opposite sides of the tubular scaffolds, the physicochemical and biological properties observed in the present study suggest that these composite grafts are inspiring candidates for ACLGs osteointegration and bone tissue regeneration.

## Figures and Tables

**Table 1:** List of primers for the osteogenic genes of interest.

Gene	Sequences		Tm (°C)
	Forward (5′–3′)	Reverse (5′–3′)	
GAPDH	ACAGTCAGCCGCATCTTCTT	GACAAGCTTCCCGTTCTCAG	58.4
Col Iα	CGAAGACATCCCACCAATCAC	GTCACAGATCACGTCATCCGC	59.6
ALP	CTCCTCGGAAGACACTCTG	AGACTGCGCCTGGTAGTTG	60.0
OPN	CCCACAGACCCTTCCAAGTA	GGGGACAACTGGAGTGAAAA	58.4
OCN	GTGCAGAGTCCAGCAAAGG	TCAGCCACTCGTCACAGC	59.4
Runx2	TTCAGACCAGCAGCACTC	CAGCGTCAACACCATCATTC	58.1
BSP	ACTGAGCCTGTGTCTTGAAA	CTTCCAACAGCCAATCACTG	56.2
BMP-2	TGAATCAGAATCCAAGCAGG	TCTTTTGTGGAGAGGATGCC	56.3

Col Iα: Collagen type Iα; ALP: Alkaline Phosphatase; OPN: Osteopontin; OCN: Osteocalcin; Runx-2: Runt-related transcription factor-2; BSP: Bone sialoprotein; BMP-2: Bone morphogenetic protein-2.

## Supplementary Materials

The following supporting information can be downloaded at: https://www.mdpi.com/article/10.3390/pharmaceutics14040697/s1, Figure S1: Representative stress–strain plot of the CTGs and PTGs measured in hydrated state. Figure S2: Degradation profile and in vitro bioactivity. (a) Weight loss of the CTGs and PTGs immersed in protease XIV (0.2 U/mL) solution for a period of 21 days (% weight). (b) Representative SEM micrographs and EDS elemental analysis of the CTGs and PTGs after 15 days of immersion in SBF solution. σ stands for standard deviation.; Figure S3: Alizarin red staining performed directly to the CTGs and PTGs after 7 and 14 days of culture. Control samples represent the stained CTGs and PTGs without cells.
