# Supplementary material for: Bioinspired Silk Fibroin-Based Composite Grafts as Bone Tunnel Fillers for Anterior Cruciate Ligament Reconstruction
Source: Pharmaceutics. 2022 Mar 24;14(4):697. doi: 10.3390/pharmaceutics14040697 (PMC9029049; doi:10.3390/pharmaceutics14040697)
Supplement: Supplementary file 1 [file pharmaceutics-14-00697-s001.zip › pharmaceutics-1621421-supplementary.pdf]

pharmaceutics

Supplementary Materials: Bioinspired Silk Fibroin-Based Composite Grafts as Bone Tunnel Fillers for Anterior Cruciate Ligament Reconstruction

Viviana P. Ribeiro, João B. Costa, Sofia M. Carneiro, Sandra Pina, Ana C.A. Veloso, Rui L. Reis and Joaquim M. Oliveira

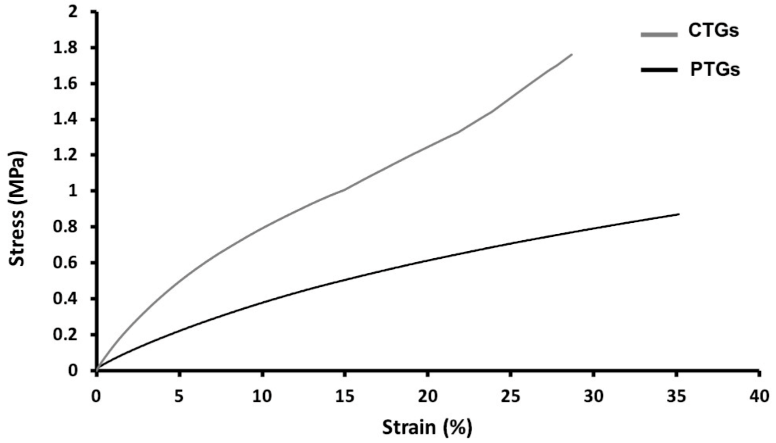

Figure S1. Representative stress-strain plot of the CTGs and PTGs measured in hydrated state.

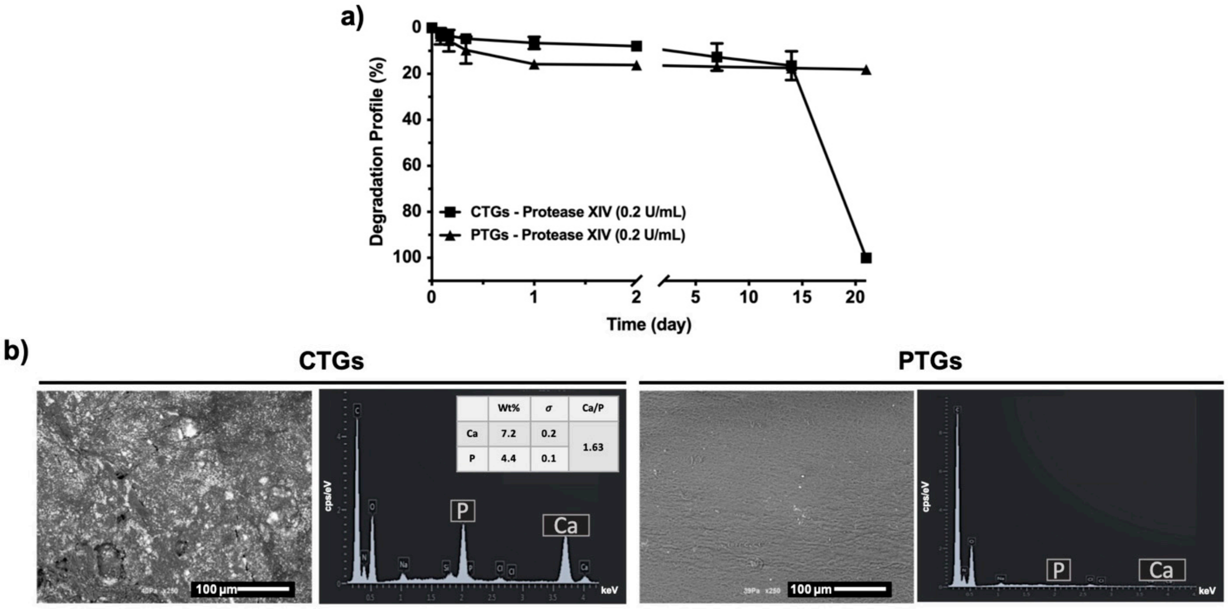

Figure S2. Degradation profile and in vitro bioactivity. (a) Weight loss of the CTGs and PTGs immersed in protease XIV (0.2 U/mL) solution for a period of 21 days (% weight). (b) Representative SEM micrographs and EDS elemental analysis of the CTGs and PTGs after 15 days of immersion in SBF solution. σ stands for standard deviation.

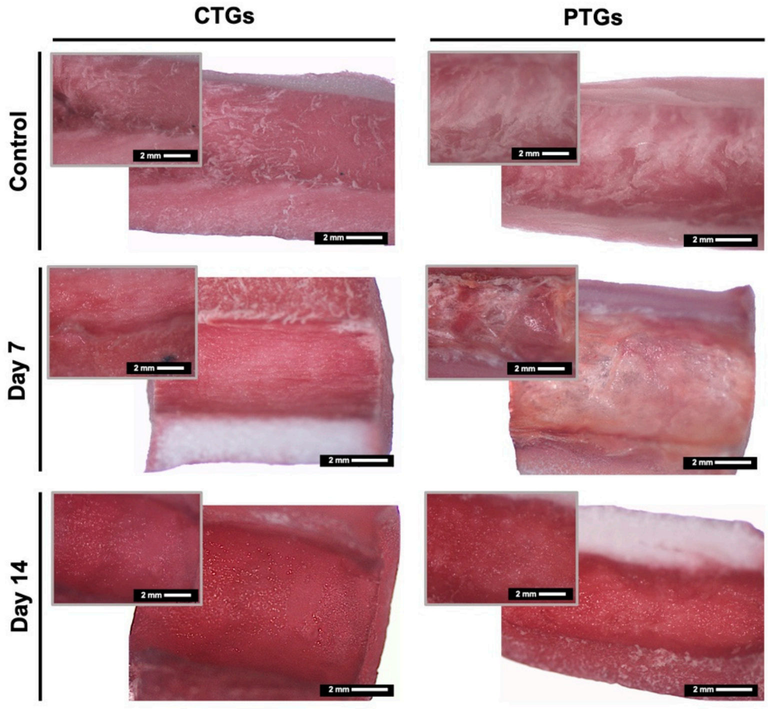

Figure S3. Alizarin red staining performed directly to the CTGs and PTGs after 7 and 14 days of culture. Control samples represent the stained CTGs and PTGs without cells.
